# Therapeutic Drug Monitoring and Pharmacokinetics-Based Individualization of Drug Therapy

**DOI:** 10.3390/pharmaceutics16060792

**Published:** 2024-06-11

**Authors:** Gellert Balazs Karvaly, Barna Vásárhelyi

**Affiliations:** Department of Laboratory Medicine, Faculty of Medicine, Semmelweis University, 4 Nagyvárad Square, 1089 Budapest, Hungary; vasarhelyi.barna@semmelweis.hu

## 1. Introduction

The philosophy, practice, and clinical impact of therapeutic drug monitoring (TDM) has changed profoundly with the appearance of widely available and, in a technical sense, commonly applicable modeling, simulation, and dosing software tools in the past decade. As a result, TDM, once a marginal field of clinical chemistry, has grown into a multidisciplinary branch of clinical medicine, allowing laboratory and pharmacometrics professionals to deliver highly relevant clinical information for supporting the management of pharmacotherapy. The newborn discipline has been given the name “model-informed precision dosing”, and is progressing to become a game changer in medicine as it lends itself to being boosted in performance and utility by machine learning and artificial intelligence tools. The eventual scope of the revolution model-informed precision dosing is bringing about is to implement individualized, patient-centric treatments [1].

TDM-guided therapies are most promising to patients with outstanding vulnerability and with atypical properties in terms of the pharmacokinetic fate of the drugs administered. Examples include oncology patients, the critically ill, those receiving donated organs, or those who have undergone major surgery. In addition, entire populations, such as children or the obese, must also be viewed as subjects to special dosing strategies. Importantly, people who are different from the majority of patients due to having atypical biological features, such as a pharmacogenetic mutation or a liver disease, will also require individual therapeutic approaches.

The nineteen research articles and the five review papers appearing in this Special Issue cover vulnerable patient populations, unique scenarios, and novel methodologies to demonstrate the utility of TDM and the pharmacokinetic processing of measurement results. Several works focus on improving the administration of anti-infective agents. On this list, one can find novel antibiotics (cefiderocol and dalbavancin) as well as ones employed globally (gentamicin, piperacillin, rifampicin, tobramycin, and vancomycin). Treatment with the antifungal isavuconazole, as well as with the antivirals ganciclovir and the cabotegravir–rilpivirine combination, are discussed. Articles focusing on immunosuppressant therapy, which is ever more often based on TDM, explore the clinical pharmacokinetics of busulfan, methotrexate, mycophenolate, and tacrolimus. Other entities, mentioned less often in the related literature, include haloperidol, omeprazole, and the combination of the antidiabetics dapagliflozin and linagliptin. Finally, this volume contains five review papers related to four different fields of clinical medicine.

## 2. Overview of the Published Works

### 2.1. Immunosuppressants

The optimization of tacrolimus dosing regimens is one of the core areas of TDM and pharmacokinetics-based treatment. Contribution (1) demonstrated the applicability of this approach in patients undergoing heart transplants. This study spans a year after receiving the organ and delivers in-depth information on the relationship between tacrolimus levels measured in whole blood and endomyocardial biopsies. An important conclusion from this study is that tacrolimus concentrations attained in the target graft organ cannot be safely estimated by determining systemic drug levels.

Methotrexate is widely administered as an immunosuppressant in pediatric oncology for the treatment of acute lymphoblastic leukemia. The remarkable interindividual variability in its pharmacokinetic properties merits model-informed precision dosing for optimized treatments. In contribution (2), six population pharmacokinetic models, published earlier, are compared with an independent external evaluation patient dataset compiled by the authors. The message obtained from this study is that pharmacokinetic models should be validated by each healthcare facility prior to their adoption.

In a retrospective monocentric study, the impact of the co-administration of calcineurin inhibitors on the twelve-hour area under the concentration–time curve of mycophenolic acid, its primary pharmacokinetic marker related to clinical efficacy, is investigated in 21 adults who have undergone a heart transplant. The follow-up had lasted at least 12 months following the transplant and the beginning of mycophenolate mofetil therapy. Exposure to mycophenolic acid, as well as its peak and trough concentrations, have been considerably lower in cases where acute cellular rejection (ACR) of the implanted organ has occurred. In addition, in comparison to the co-administration of tacrolimus, co-medication with cyclosporine A has resulted in a substantially higher proportion of cases with ACR (contribution (3)).

### 2.2. Anti-Infectives

The clinical pharmacokinetics of ganciclovir have been explored in patients diagnosed with cystic fibrosis (CF) who have undergone lung transplants using Bayesian modeling, with the findings being compared to those observed in non-cystic fibrosis patients. The clearance of this antiviral appears to be higher in the CF population, leading to lower systemic exposure. Nomograms are provided for administering loading and maintenance doses by taking the height of the patients and the estimated glomerular filtration rates into account (contribution (4)).

The preliminary pharmacokinetic results of a nationwide multicenter study with the scope of following up HIV-positive patients receiving a combined long-acting injectable formulation of cabotegravir–rilpivirine by therapeutic drug monitoring are presented in contribution (5). This work was the first to test pharmacokinetic models for the real patient population, with the aim of defining a trough therapeutic range.

Isavuconazole is a relatively new agent effective against systemic fungal infections. It was approved for the treatment of invasive aspergillosis and invasive mucormyosis in December 2023, but, prior to that date, it had been used off-label for the treatment of immunocompromised pediatric patients. Contribution (6) described the retrospective pharmacokinetic evaluation of the TDM program of isavuconazole conducted in a pediatric hospital and offers a methodology for the optimized, model-informed precision dosing of isavuconazole in this especially vulnerable patient population.

Cefiderocol is a new cephalosporin active against Gram-negative strains, including those producing beta-lactamase and carbapenemase. The authors of contribution (7) investigated its population pharmacokinetics in critically ill adults for the first time. They revealed that serum albumin concentration and chronic kidney disease are predictors of the proportion of the unbound fraction of cefiderocol in the circulation.

A novel approach to estimating individual piperacillin concentrations by constructing nonparametric artificial population pharmacokinetic (PK) quasi-models is described in contribution (8). The number of volunteers included in a population pharmacokinetic study is often low, which results in limited utility, including the comparability of findings. The authors of this study demonstrated that the quasi-models are efficient for augmenting population PK models and could provide a tool for generating individual concentration estimates when the population PK model has been obtained by including data from a small set of subjects.

The efficient guidance of vancomycin dosing is an especially complex task in pediatric patients, considering the variability of the physiological properties of different age groups and of various individuals in each age group. The authors of contribution (9) presented population pharmacokinetic models by evaluating renal function using urinary and blood cystatin C and neutrophil gelatinase-associated lipocalin (NGAL) concentrations. Three modeling approaches were compared in this study, and it was demonstrated that their performance cannot be associated with the complexity of the model, favoring the consideration of simpler models for clinical use.

Dalbavancin is one of the very few new entities available for the treatment of serious Gram-positive bacterial infections. It has an unusual pharmacokinetic profile, including an elimination half-life of over 400 h. The strategy of its administration, as well as the monitoring of the course of therapy, is substantially different from that applied to most drugs. In contribution (10), the associations and correlations of four dosing regimens with the characteristics of patients, the pathogen, and the clinical situation are discussed, including the analysis of the relationship between individual dalbavancin pharmacokinetics and therapeutic outcomes.

In contribution (11), the authors demonstrate that the implementation of model-informed precision dosing of tobramycin for treating pulmonary exacerbations caused by *Pseudomonas aeruginosa* in cystic fibrosis results in the administration of higher doses and is also associated with success in therapy. They also show that, in spite of dose elevations, renal function improves by the end of the treatment.

The external validation of population pharmacokinetic models may not necessarily demonstrate their broader utility. For such cases, the re-estimation of gentamicin model parameters is proposed in contribution (12). Four parametric models, published earlier, are applied to two datasets obtained by the authors in critically ill populations in the framework of TDM conducted at their facility. It was shown that none of them is capable of making acceptable predictions of pharmacokinetic–pharmacodynamic targets, but their performance is improved substantially when model parameters are re-estimated using the new datasets. In the future, this re-estimation strategy may prevent the construction of redundant models, with the incorporation of new data increasing the robustness of existing ones.

An account was given by contribution (13) concerning treatment with 10 mg/kg rifampicin based on TDM to take initiative against the high prevalence of tuberculosis in native Paraguayans in comparison to non-natives. In this study, a realistic procedure was elaborated for collecting dried blood samples from tuberculosis patients across the country. The probabilities of pharmacokinetic–pharmacodynamic target attainment were calculated. When individual minimal inhibitory concentration (MIC) values were not available, the attainment of targets depended solely on the hypothetical MIC considered. The authors of this study concluded that administering an increased dose (35 mg/kg) of rifampicin warrants investigation for improving outcomes.

### 2.3. Miscellaneous Drugs

Contribution (14) is the first clinical study to describe the pharmacokinetics of busulfan in patients diagnosed with neurofibromatosis, a condition that can currently be cured only by hematopoietic stem cell transplantation. This was also the first effort to include the metabolite sulfolane in a busulfan population pharmacokinetic model. Total body weight was identified as the main covariate. The findings were expected to allow the individualized dosing of busulfan to neurofibromatosis patients, restricting the development of irreversible liver impairment.

Anticoagulant therapy has changed dramatically since the introduction of direct-acting oral anticoagulants. Although the relationship between the pharmacokinetics and the pharmacodynamic effects of these drugs is still unclear, it is now understood that the interindividual variability of exposure is high and overexposure may cause adverse effects. Population pharmacokinetic models based on real-life therapeutic drug monitoring as well as simulations are presented in contribution (15) to compare the exposure to rivaroxaban, administered to Thai adults diagnosed with non-valvular atrial fibrillation based on renal function, or in standard dosages. It is shown that in this population, renal function and body weight are covariates, and the prescription of standard 20 mg dosages will likely lead to overexposure.

The impact of C-reactive protein (CRP) on exposure to haloperidol following the administration of low haloperidol doses in critically ill adults was described in contribution (16). By constructing its parametric Bayesian model and by performing Monte Carlo simulations, the authors showed that the clearance of haloperidol is negatively correlated with increasing CRP up to approximately 50 mg/L, pointing to the utility of individualized dosing at the intensive care unit to overcome delirium.

In contribution (17), the pharmacokinetics and safety of the antidiabetics dapagliflozin and linagliptin, given as an experimental combination product formula, were compared to those recorded during the separate administration of the two substances in a randomized, open-label, single-dose study conducted with healthy male volunteers. The bioequivalence of the two formulations was demonstrated, providing important evidence that the combined administration of these pharmacologically complementary entities is a rational, patient-centric approach.

Gastrectomy can alter the absorption of drugs from the gastrointestinal system profoundly. Laparoscopic sleeve gastric surgery (LSG), a widely employed bariatric intervention, leads to a considerable reduction in gastric capacity and, as a result, increased acid reflux, which is countered by the prolonged administration of proton pump inhibitors (PPIs). In contribution (18), it is shown that the faster absorption of omeprazole, a well-known PPI, takes place after surgery has been performed. The results of the comparison of omeprazole exposure among normal, intermediate, and poor cytochrome P2C19 metabolizer obese patients who have undergone LSG are also presented. The authors concluded that 20 mg of omeprazole given once daily is sufficient for this population.

One of the key advantages of pharmacokinetics-based individualized drug therapy is that, given a population pharmacokinetic model relying on rich data, individual pharmacokinetic information can be inferred by measuring drug concentrations in a small number of samples (referred to as a *limited sampling strategy*). An approach is provided in contribution (19) to evaluating the robustness of such strategies by assessing the elimination of iohexol, a gold standard in characterizing glomerular filtration rates, from the circulation by enforcing pre-planned deviations from planned sampling times.

### 2.4. Reviews

In this work, timely topics related to cystic fibrosis, oncology, ophthalmology, and psychiatry are reviewed. Westra et al. discussed pharmacological approaches to increasing exposure to protein kinase inhibitors (contribution (20)). In another article, an overview is provided of current knowledge regarding the therapeutic monitoring of these substances in peripheral fluid spaces (contribution 21). Comprehensive updates are presented on the monitoring of cystic fibrosis transmembrane conductance regulators (CFTCRs) as well as of biological therapy substances applicable against non-infectious uveitis in contributions (22) and (23). Finally, a review of the most recent literature of TDM in psychiatry, starting with the publication of the latest update of the widely accepted guidelines of the Arbeitsgemeinschaft fur Neuropsychopharmakologie und Pharmakopsychiatrie, is delivered [2].

## 3. Future Perspectives

Claiming that we have entered a golden era of TDM is perhaps not an exaggeration. The diversity and outstanding importance of the topics, the multitude of software employed, and the long road that lies ahead in implementing the approaches described clearly highlight the perspectives of this exciting and versatile field. It is also evident that multidisciplinarity is of key significance; the structures, procedures, and infrastructure maintaining the efficiency of the multidisciplinary clinical teams must be elaborated and continuously improved to support the translation of the knowledge that emerges into clinical protocols. These challenges raise a plethora of scientific questions, which will certainly keep the topics of therapeutic drug monitoring and pharmacokinetics-based individualized treatment current for years to come.
